# Multiple sclerosis diagnosis and its differential diagnosis in patients presenting with type four ‘mirror pattern’ CSF oligoclonal bands

**DOI:** 10.1007/s00415-025-12947-y

**Published:** 2025-02-15

**Authors:** Damiano Marastoni, Monica Sicchieri, Francesca B. Pizzini, Arianna Scartezzini, Federica Virla, Ermanna Turano, Daniela Anni, Maddalena Bertolazzo, Stefano Ziccardi, Valentina Camera, Agnese Tamanti, Maddalena Marini, Giuseppe Lippi, Bruno Bonetti, Andrew J. Solomon, Massimiliano Calabrese

**Affiliations:** 1 Neurology B, Department of Neurosciences, Biomedicine and Movement, University of Verona, Policlinico “G.B. Rossi” Borgo Roma, Piazzale L. A. Scuro, 10, 37134 Verona, Italy; 2 Radiology and Neuroradiology Unit, Azienda Ospedaliera Universitaria Integrata, Verona, Italy; 3 Section of Clinical Biochemistry, University of Verona, Verona, Italy; 4 Neurology A, Azienda Ospedaliera Universitaria Integrata Di Verona, Verona, Italy; 5 Department of Neurological Sciences, Larner College of Medicine at the University of Vermont, Burlington, VT USA

**Keywords:** Multiple sclerosis diagnosis, Cerebrospinal fluid, Oligoclonal bands, Mirror pattern, Differential diagnosis

## Abstract

**Background:**

Presence of oligoclonal bands (OCBs) restricted to cerebrospinal fluid (CSF) characterizes most patients with multiple sclerosis (MS). Few data are available on the frequency of MS diagnosis and the main alternative diagnoses in patients with an initial central nervous system (CNS) demyelinating event and CSF IV pattern, the so-called ‘mirror pattern’.

**Methods:**

Seventy-six patients presenting with OCBs pattern IV after a clinical attack suggestive of CNS demyelinating event were included in the study. Diagnostic work-up, including blood, CSF, and paraclinical examinations, and 2 years of clinical and radiological follow-up were evaluated.

**Results:**

Pattern IV occurred in 15.1% of patients. Twenty-five patients (32.8%) received a diagnosis of MS, thirty-two (42.1%) an alternative diagnosis, and nineteen (25%) remained without definite diagnosis. Most frequent alternative diagnosis was encephalopathy with atypical MRI lesions of probable vascular origin** (**19.7%). MS was significantly more common in patients with type IV OCB pattern (25 of 76) than in a group of patients presenting with type I OCB pattern (32 of 168, *p* = 0.017).

**Conclusion:**

The diagnosis of MS is common in patients who present with OCBs pattern IV. However, other CNS disorders, particularly vascular encephalopathy, should be carefully considered.

## Introduction

For decades, IgG oligoclonal bands (OCBs) restricted to CSF have been recognized as a hallmark of multiple sclerosis (MS) [1]. The latest revision of the McDonald criteria incorporates OCBs in the diagnostic process for MS, allowing accurate and earlier diagnosis in patients with monophasic syndromes lacking demonstration of dissemination in time [2]. According to European Consensus guidelines, isoelectric focusing electrophoresis (IEF) is the most sensitive method to detect OCBs [3, 4]. Five IEF patterns have been described: i) type 1, the usual pattern, with the polyclonal IgG distribution in both CSF and serum; ii) type 2: OCBs present only in CSF; iii) type 3: OCBs confined to CSF with additional and identical bands in CSF and serum; iv) type 4 (‘mirror pattern’): identical OCBs in CSF and serum, no indication of local synthesis; v) type 5: monoclonal bands in CSF and serum [5].

The presence of CSF-restricted OCBs (patterns 2 and 3) has high sensitivity and specificity for a diagnosis of MS compared with other CNS inflammatory disorders, particularly in patients with a typical first demyelinating attack [6]. In contrast, the absence of CNS-restricted OCBs has been associated with misdiagnosis of MS [7, 8], even though approximately 10% of MS patients are diagnosed with ‘OCB negative’ MS [9].

Despite revisions to diagnostic criteria for MS that have demonstrated improved sensitivity and specificity, in some patients, the diagnosis of MS often remains difficult. Several paraclinical tools have shown promise to aid accurate diagnosis in such cases: these include some MRI characteristics such as the presence of the central vein sign, cortical lesions and paramagnetic rim lesions, but also OCT [7, 10–13]. By comparison, there have been few investigations concerning IEF patterns in patients in the differential diagnosis of MS. In particular, pattern IV is an uncommon outcome of IEF in patients with the first demyelinating event [14]. To our knowledge, no data are available on (i) the risk of having MS in these patients, particularly compared to other OCB patterns; (ii) alternative diagnoses in this group of patients. The aim of this study was to characterize diagnoses in patients presenting for evaluation for MS who were found to have pattern IV OCBs.

## Materials and methods

### Patient cohort

In this longitudinal observational study, we systematically investigated clinical and laboratory reports of patients showing OCB IV pattern at the initial diagnostic evaluation.

This study is part of a larger study that included 502 consecutive patients aimed to study the link between the CSF analysis and the MRI profile of patients evaluated after an initial demyelinating attack.

The other inclusion criteria were:

- A first clinical attack suggestive of a CNS demyelinating disorder that led to a neurological referral to the MS Center of the Azienda Ospedaliera Universitaria of Verona, Italy, between January 2017 and January 2022;
- Availability of a complete diagnostic work-up, including blood and CSF examinations, brain and spinal cord MRI, to assess the diagnosis of MS;
- At least 2 years of clinical and radiological follow-up.

### Study design

**Fig. 1:**
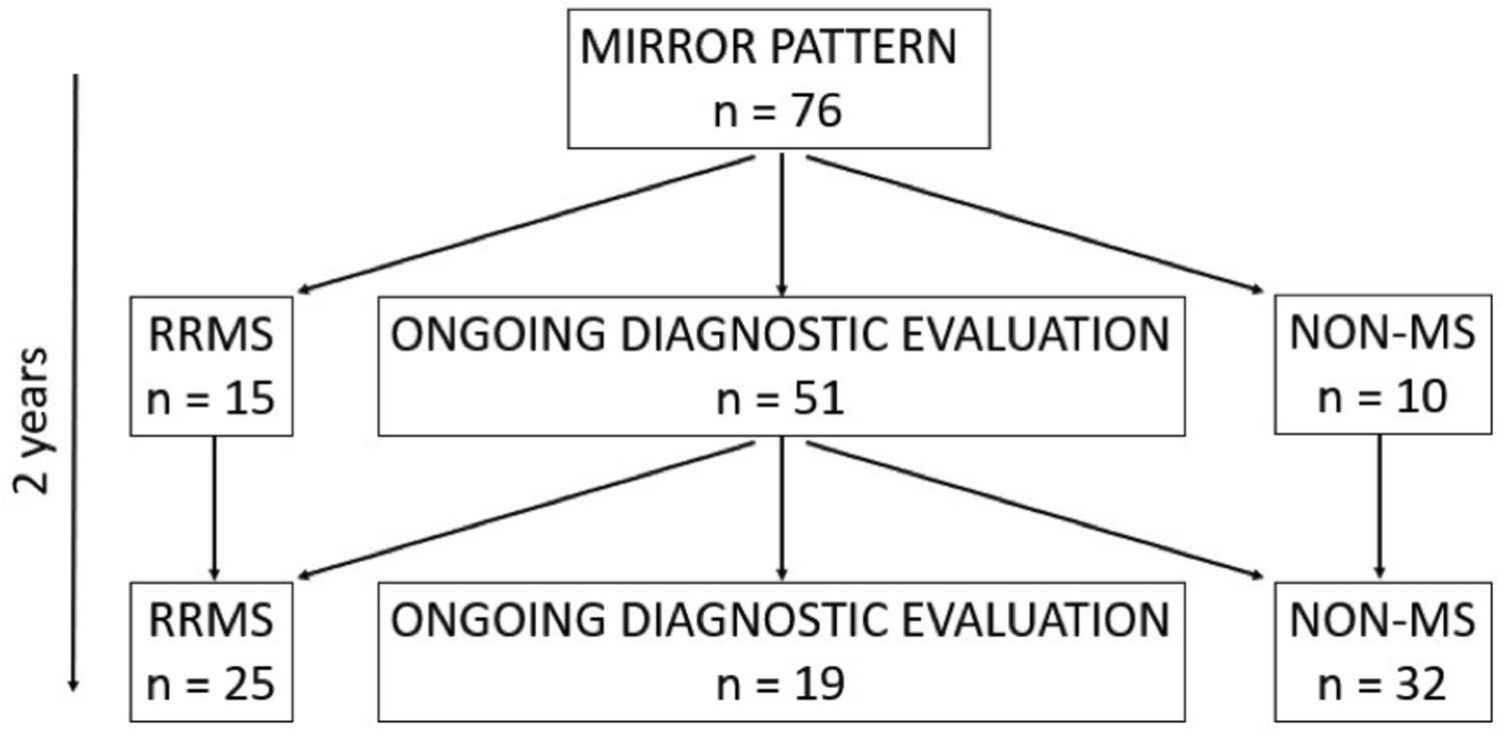
Study plan. Patients’ cohort according to the diagnosis received at initial diagnostic work-up and after 2-year follow-up. *RRMS* relapsing–remitting multiple sclerosis

Each patient underwent a full diagnostic work-up at the time of the first demyelinating event. In case a conclusive diagnosis was not reached, patients underwent a 2-year clinical and radiological follow-up. Both at the end of the first diagnostic work-up and after 2 years of follow-up, all recruited patients were classified into three groups according to diagnosis: (i) MS according to the 2017 McDonald criteria [2]; (ii) non-MS (indication of an alternative diagnosis was required); (iii) ongoing diagnostic evaluation: conclusive diagnosis could not be made and further follow-up was required. The flowchart of the study is summarized in Fig. 1.

Each alternative diagnosis was based on the most recent international guidelines where applicable (i.e., migraine, Myelin Oligodendrocyte Glycoprotein Antibody-Associated Disease-MOGAD, neuromyelitis optica spectrum disorder [NMOSD], Sjogren syndrome, systemic lupus erythematosus) or clinical judgment where established diagnostic guidelines are lacking.

### Clinical evaluation

All patients underwent clinical evaluations every 6 months, with additional visits in the case of new symptoms for relapse assessment. The site of clinical onset was recorded for each patient. Disability was assessed using the Expanded Disability Status Scale (EDSS) score [15]. A relapse was defined as a worsening of neurological impairment or the appearance of a new symptom or abnormality attributable to MS, lasting at least 24 h, in the absence of fever, and preceded by stability of at least 1 month [16]. The appearance of new or enlarging T2 lesions or gadolinium-enhancing lesions defined MRI activity. Finally, the occurrence of comorbidities was assessed.

### Evaluation of OCBs and other paraclinical assessments

OCB pattern [3] was evaluated by isoelectric focusing and agarose gel immunofixation [17].

The minimum blood laboratory evaluation required at the initial work-up included a complete blood count, AST, ALT, gGT, renal profile, ANA, ENA, antiphospholipid antibodies, antithyroid antibodies, AQP4- IgG and MOG-IgG, homocysteine, vitamin B12, folate, ESR, CRP, urine examination. Infections, including HIV and syphilis, were excluded by blood analysis.

All patients underwent brain MRI with gadolinium. The size, morphology, and location of each MRI lesion were evaluated. Oval, asymmetric white matter lesions perpendicular to the ventricles (Dawson’s fingers) or lesions in the periventricular, juxtacortical, infratentorial, or spinal cord region, with a diameter > 3 mm, were considered typical of MS according to the practical guidelines for evaluating lesions on MRI [18–20]. We also assessed the number of contrast-enhancing lesions and fulfillment of dissemination in the space.

Visual evoked potentials (VEP) were performed in a subgroup of patients following the recommendations of the International Society for Clinical Electrophysiology of Vision [21], and latency and morphology were recorded.

### Statistical analysis

Differences between groups were initially assessed using the Mann–Whitney and Chi-square/Fisher exact tests when appropriate. The χ2 test was applied to test for the effect of MRI lesion type, VEP abnormalities, spinal cord lesions, and gadolinium-enhancing lesions in identifying MS patients versus patients who did not receive a diagnosis of MS. Finally, χ2 test was used to test the difference in the frequency of MS between a comparison cohort of patients with OCB pattern I and patients without OCBs IV pattern.

### Ethics approval

The local Ethics Committee approved the study, and all patients signed an informed consent (MSBioB, 2413CESC).

### Data availability statement

Deidentified data will be shared upon request by qualified researchers.

## Results

Among the 502 patients evaluated, 76 patients (15.1%) with the OCB IV pattern met the inclusion criteria (Fig. 1).

**Fig. 2:**
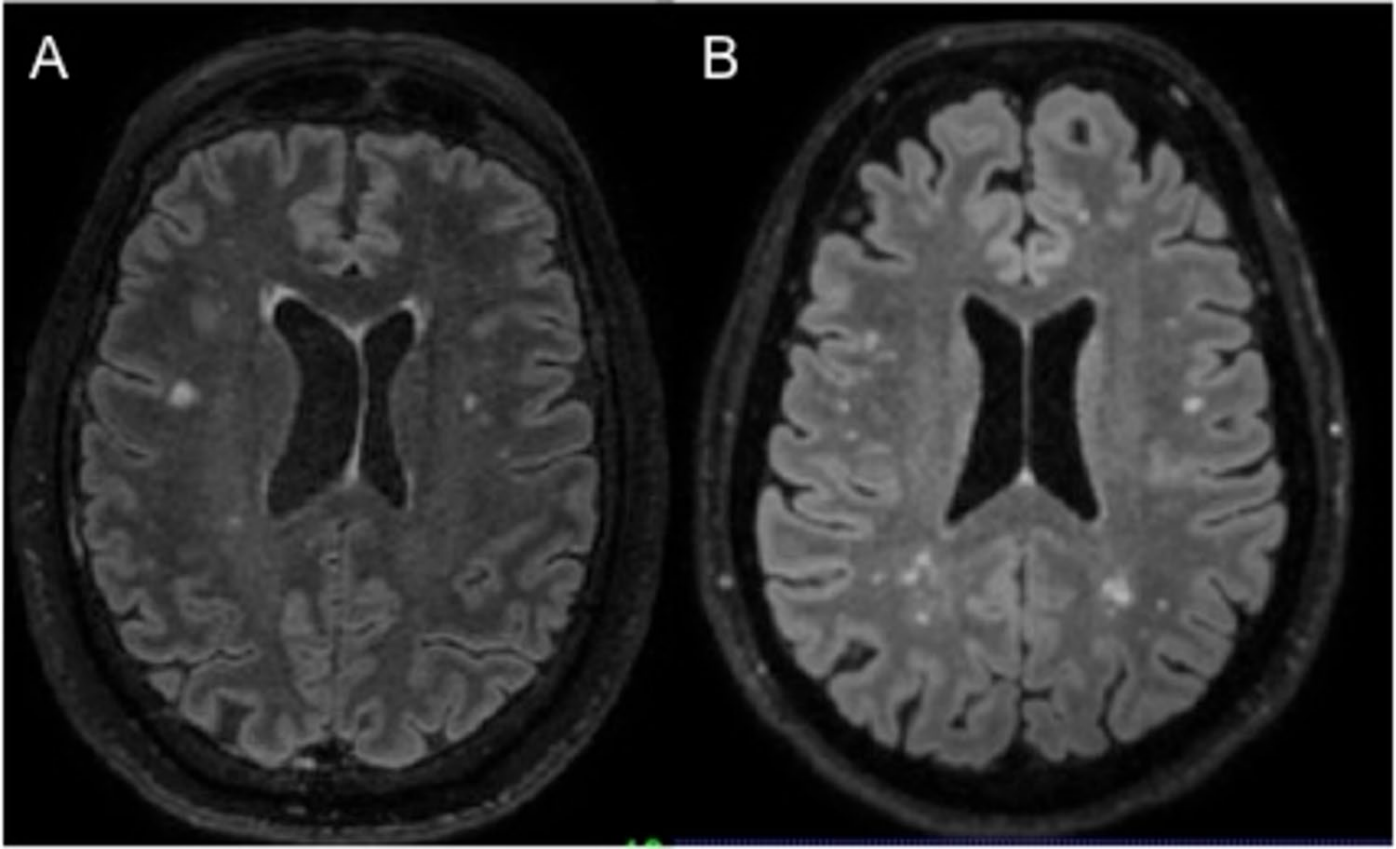
Atypical MRI lesions in patients with an alternative diagnosis. **a** Bilateral small juxtacortical frontal T2-hyperintese lesions, the larger one within the right inferior frontal gyrus (5 mm longitudinal axis) in a patient with anti-MOG encephalopathy. **b** Bilateral small juxtacortical, deep and periventricular frontal parietal and T2-hyperintese lesions, partially confluent within the posterior white matter in a patient with vascular encephalopathy

Fifteen (19.7%) received a diagnosis of RRMS immediately after the initial diagnostic work-up (Table 1), while ten received an alternative diagnosis (Fig. 2, Table 2). In 51 cases, inconclusive diagnosis required further clinical and radiological follow-up.

**Table 1:** Patient cohort characteristics at baseline and after 2 years of follow-up

	Total	MS	Non-MS	Diagnostic evaluation ongoing	*p*
At the initial diagnostic evaluation					
* N*	76	15	10	51	
Age of onset (years, mean ± SD)	43.1 ± 14.3	33.6 ± 12.5	45.6 ± 12.2	45.4 ± 14.1	**0.031**
Gender (f:m)	49:27	11:4	5:5	33:18	0.397
EDSS (median, range)	1 (0–6)	1.5 (0–4)	1.5(0–3)	1 (0–6)	0.977
Type of onset					
- Cerebral (%)		4 (26.7)	5 (50)	19 (37.3)	0.397
- Spinal (%)		3 (20)	3 (30)	19 (37.3)	0.653
- Cerebellar (%)		2 (13.3)	0 (0)	1 (2)	0.500
- Brainstem (%)		3 (20)	1 (10)	8 (15.7)	0.627
- Optic nerve (%)		3 (20)	1 (10)	4 (7.8)	0.627
After 2 years of follow-up					
*N*	76	25	32	19	
Age of onset (years, mean ± SD)	43.1 ± 14.3	36.8 ± 13.8	46.2 ± 12.6	44.9 ± 14.6	**0.015**
Gender (f:m)	49:27	17:8	20:12	12:7	0.782
EDSS (median, range)	2 (0–6.5)	2.5 (0–7)	1(0–6.5)	1(0–6)	**0.029**
Type of onset					
- Cerebral (%)		8 (32)	17 (53.1)	5 (26.3)	0.178
- Spinal (%)		3 (12)	8 (25)	11 (57.9)	0.315
- Cerebellar (%)		3 (12)	1 (3.1)	0 (0)	0.309
- Brainstem (%)		5 (20)	5 (15.6)	2 (10.5)	0.735
- Optic nerve (%)		6 (24)	1 (3.1)	1 (5.3)	**0.036**

After 2 years of follow-up, patients with MS were younger and showed greater disability than the ‘non-MS’ group

*p* value indicates comparisons between patients with MS and the ‘non-MS’ group of patients

**Table 2:** Diagnosis after the initial diagnostic evaluation and after 2 years of follow-up

After initial diagnostic evaluation	After 2 years of follow-up
**RRMS: 15/76 (19.7%)**	**RRMS: 25/76 (32.9%)**
**Diagnostic evaluation ongoing: 51/76 (67.1%)**	**Diagnostic evaluation ongoing: 19/76 (25.0%)**
**NON-MS: 10/76 (13.2%)**	**NON-MS: 32/76 (42.1%)**
MOGAD: 3 (3.9%)	Encephalopathy with atypical MRI lesions of probable
Compressive myelopathy: 2 (2.6%)	vascular origin: 15 (19.7%)
Vascular encephalopathy: 1 (1.3%)	MOGAD: 3 (3.9%)
Systemic lupus erythematosus: 1 (1.3%)	Sjogren syndrome: 2 (2.6%)
Sjogren syndrome: 1 (1.3%)	Migraine: 2 (2.6%)
Idiopathic VII nerve palsy: 1 (1.3%)	Non-Hodgkin lymphoma: 2 (2.6%)
Lymphocytic encephalitis: 1 (1.3%)	Compressive myelopathy: 2 (2.6%)
	CNS vasculitis: 1 (1.3%)
	CNS vasculitis: 1 (1.3%)
	CNS vasculitis: 1 (1.3%)
	Antiganglioside antibodies neuropathy: 1 (1.3%)
	Polycythemia vera: 1 (1.3%)
	Polycythemia vera: 1 (1.3%)
	Polycythemia vera: 1 (1.3%)
	Idiopathic VII nerve palsy: 1 (1.3%)
	Idiopathic VII nerve palsy: 1 (1.3%)
	Lymphocytic encephalitis: 1 (1.3%)
	Systemic lupus erythematosus: 1 (1.3%)

Among the 51 patients with an ongoing diagnostic investigation at the initial diagnostic evaluation, 10 received a diagnosis of MS, 14 of encephalopathy with atypical MRI lesions of probable vascular origin and 8 received an alternative diagnosis (Table 2).

MS patients were younger (mean age 36.8 ± 13.8 years) than patients with an alternative diagnosis (mean age 46.2 ± 12.6 years, *p* = 0.015) and showed greater disability (median EDSS 2.5 [0–7] vs 1 [0–6.5], *p* = 0.029). Diagnoses at initial work-up and after 2 years are shown in Table 2. Table 3 details how patients fulfilled the diagnostic criteria for MS.

**Table 3:** How patients with a mirror pattern achieved a diagnosis of MS according to 2017 McDonald Criteria revision

	MS at initial diagnostic work-up (*n = *15)	MS after 2 years of follow-up (*n = *10)
Type of clinical onset	- Cerebral: 2 - Spinal: 3 - Cerebellar: 4 - Brainstem: 3 - Optic nerve: 3	- Cerebral: 0 - Spinal: 4 - Cerebellar: 1 - Brainstem: 2 - Optic nerve: 3
Number of lesions with objective clinical evidence	- 2 lesions: 3 - 1 lesion: 12	**-** 1 lesion: 10
DIS	- > 2 attacks: 2	- 1 attack: 10
- Clinical attacks	- 2 attacks: 5	- additional clinical attack
	- 1 attack: 8	implicating a different CNS site: 4
- MRI demonstration	- MRI demonstration: 15	- MRI demonstration: 6
DIT	- > 2 attacks: 2	- 1 attack: 10
- Clinical attacks	- 2 attacks: 5	- new clinical attacks: 4
	- 1 attack: 8	
- MRI demonstration	- Gadolinium	- new T2 MRI lesions: 6
	enhancing lesions: 11	

*MS*, multiple sclerosis, *DIS* dissemination in space, *DIT* dissemination in time, *CNS*, central nervous system

In the MS group with ‘mirror pattern’, 37.5% of patients had abnormal visual evoked potentials, without a previously reported optic neuritis. Spinal cord lesions were detected in 68.4% of cases, and gadolinium enhancement was present in 58.8%. Of these, only 8% had atypical MRI lesions. In contrast, atypical MRI lesions were common in the ‘non-MS’ group (23/51, 45.1%, *p* = 0.002, Table 4).

**Table 4:** Paraclinical assessment in the MS and non-MS cohorts after the 2-year follow-up

Paraclinical assessments	Total	MS (*n = *25)	Not MS/diagnostic evaluation ongoing (*n = *51)	*P*
Gd + lesions	24/54 (44.4%)	10/17 (58.8%)	14/37 (37.8%)	0.238
Spinal cord lesions	33/63 (52.4%)	13/19 (68.4%)	20/44 (45.45%)	0.109
Abnormal PEVs	10/30 (33.3%)	3/8 (37.5%)	7/22 (31.8%)	0.999
Atypical MRI lesions	25/72 (34.7%)	2/25 (8%)	23/51 (45.1%)	**0.002**

The presence of atypical MRI lesions was characteristic of patients without a final diagnosis of MS

*P* value < 0.05 was considered significant. Comparisons between patients with MS and those who have not reached an MS diagnosis are shown

Comorbidities were noted in patients with type IV OCB. Comorbidities are shown in Table 5. The most common comorbidity was thyroid pathology (*n = *9, 11.8%) and rheumatic signs or symptoms were also common (*n = *9, 11.8%).

**Table 5:** Comorbidities in patients with ‘mirror pattern’

Comorbidities in patients with MS and ‘mirror pattern’ (*n = *25)	Comorbidities in patients ‘Non-MS/diagnostic evaluation ongoing’ and ‘mirror pattern’ (*n = *51)
Thyroid pathology (3 hypothyroidism, 1 hyperthyroidism): 4 (16%) Rheumatologic disorders (1 ANA + , 1 Ab anti Citrullin + , 1 Undifferentiated arthritis): 3 (12%) Favism: 1 (1.31%) Autoimmune hepatitis: 1 (1.31%) Crohn’s disease: 1 (1.31%) Type 1 diabetes mellitus: 1 (1.31%) Monoclonal gammopathy: 1 (1.31%)	Thyroid pathology (5 Hypothyroidism): 5 (9.8%) Rheumatologic disorders: (5 ANA + , 1 FR +): 6 (11.8%) Monoclonal gammopathy: 2 (3.9%) Breast cancer: 2 (3.9%) Ovarian cancer: 2 (3.9%) Migraine: 2 (3.9%) Crohn’s disease: 1 (2%) Type 1 diabetes mellitus: 1 (2%) Celiac disease: 1 (2%) Colon cancer: 1 (2%) Vitiligo: 1 (2%) HBV infection: 1 (2%) Glaucoma: 1 (2%) Psychiatric disorders: 1 (2%) Uterine cancer: 1(2%) Previous stroke: 1 (2%) Non-Hodgkin lymphoma: 1 (2%)

Among the 502 patients, 168 showed type I OCB. After a 2-year clinical follow-up, 32 (19.0%) received a final MS diagnosis (mean age 37.4 ± 11.4 years), while 136 received an alternative diagnosis (mean age 44.1 ± 10.3 years, *p* = 0.003). Encephalopathy with atypical CNS lesions of probable vascular origin (patients with small vessels disease who developed small vascular lesions atypical for MS) was the most common diagnosis (31, 18.4%). Notably, the diagnosis of MS was significantly more common in patients with a ‘mirror pattern’ (25/76) than in patients with type I OCB pattern (32/168, 19.0%, *p* = 0.017).

Finally, 258 patients presenting an initial demyelinating attack and with the presence of OCB (pattern 2 or 3) were also evaluated. Among these, 221 (85.6%) received the diagnosis of MS, while the remaining 37 are still under investigation with a diagnosis of encephalopathy with atypical CNS lesions of probable vascular origin being the most frequent diagnosis.

## Discussion

Patients with MS without CSF type II and III pattern represent approximately 10%–12% of cases [9, 14]. These cases have been associated with suspected misdiagnosis of MS [8], and OCB patterns in patients without type II and III pattern are infrequently reported in the literature.

In our cohort, in patients with type I and type IV OCB patterns, MS was diagnosed in approximately 1 in 5 and 1 in 3 patients, respectively. This observational, real-life longitudinal study, based on clinical practice, provided evidence that the occurrence of MS in those patients with a ‘mirror pattern’ is not uncommon. Furthermore, patients who experienced a first possible demyelinating event and had a IV OCB pattern showed an increased risk of MS compared to type I OCBs. Encephalopathy with atypical lesions of probable vascular origin was the most common alternative diagnosis in patients with both type IV and I patterns, supporting data from larger cohorts of patients presenting for suspected MS [7].

MS patients with a ‘mirror pattern’ were characterized by a younger age than the group without MS suggesting perhaps that advanced age is a potential ‘red flag’ in patients presenting with type IV OCBS and evaluation for MS. While type of clinical onset, other than optic neuritis, was not useful in discriminating patients with or without MS in the “mirror pattern” group, atypical MRI lesions smaller than 3 mm in their long axis and located in specific brain regions [19] were more frequent in patients with alternative diagnoses. The presence of gadolinium-enhancing lesions or spinal cord lesions was not a discriminant for the diagnosis of MS. Among paraclinical tests, VEP alterations were not different between patients who ultimately received a diagnosis of MS or not. This was partially unexpected, as abnormal VEPs are typical of MS and useful in the diagnostic process [7]. Nevertheless, the relatively low number of patients with available VEP data may have influenced our results. Ongoing research has focused on additional characteristics of MS lesions, such as the “central vein sign” [22] or the presence of a paramagnetic rim [23], but data in patients with MS and type IV OCBS are unfortunately lacking. Finally, according to the diagnoses that have been obtained in patients with IV OCB pattern, we suggest the application of an extended panel of blood examinations, with a particular focus on the exclusion of hematological and rheumatological morbidities.

There is consistent evidence that the presence of OCBs limited to the CNS is associated with increased disability. OCB + patients had a higher risk of achieving an EDSS of 6 and early disability progression [24, 25]. Furthermore, increased cortical lesion burden and increased expression of inflammatory markers have been associated with CSF-restricted oligoclonal bands in MS [26]. Unfortunately, these data are completely lacking in MS and ‘mirror pattern’ patients, thus suggesting that a more accurate CSF profiling, including proteomic analysis, of ‘mirror pattern’ patients would help in the diagnostic work-up and characterization of the risk of disability progression in these patients. Interestingly, increased disability at the initial presentation was noted in patients diagnosed with MS after 2 years of follow-up with type IV OCBS compared to patients with alternative diagnoses.

The study has a number of limitations. These include referral bias, the relatively small number of patients, and the short duration of follow-up. Furthermore, CSF analysis was not repeated, thus potentially underestimating the occurrence of OCB restriction when repeating the analysis in patients with early MS [27]. Few data are available from longitudinal studies with repeated lumbar punctures. In a cohort study, 12.5% of patients who underwent a second lumbar puncture, had changes in OCB status, with in most cases disappearance of OCBs, without a significant impact on the final diagnosis [28]. Furthermore, a repeated lumbar puncture could provide value in evaluating treatment effect on the intrathecal compartment, with in some cases in disappearance of OCBs after treatment for MS [29, 30]. We believe that consideration should be given to repeating the lumbar puncture and CSF analysis if clinical suspicion is high but results of CSF are equivocal, negative, or show only a single band [27]. Comparison of the clinical characteristics of patients with MS and type IV and type I OCB patterns to patients with MS with CSF-restricted OCBs may have been informative, but was beyond the scope of this study. Finally, the presence of a ‘mirror pattern’ cannot exclude the presence of intrathecal IgG synthesis. Accordingly, additional methods that could increase sensitivity for intrathecal humoral activity should be validated in clinical practice [31–33].

In conclusion, although pattern IV is not the most common OCB pattern in MS, in our cohort, MS was frequently diagnosed in patients with this OCB pattern. Moreover, the risk of MS was higher in patients presenting for MS evaluation with OCB pattern IV compared to pattern I, perhaps suggesting that MS in that such patients warrant careful consideration for MS while ruling out other inflammatory CNS disorders. Future studies in larger cohorts are needed to confirm these data to inform diagnostic evaluation and to clarify the natural history of MS in patients without signs of intrathecal IgG synthesis.

## Data Availability

Deidentified data will be shared on request from a qualified investigator.
